# Supplementary material for: Iron Deposition and Ferroptosis in the Spleen in a Murine Model of Acute Radiation Syndrome
Source: Int J Mol Sci. 2022 Sep 20;23(19):11029. doi: 10.3390/ijms231911029 (PMC9570444; doi:10.3390/ijms231911029)
Supplement: Supplementary file 1 [file ijms-23-11029-s001.zip › ijms-1871754-supplementary.pdf]

Supplementary materials

Sham irradiation

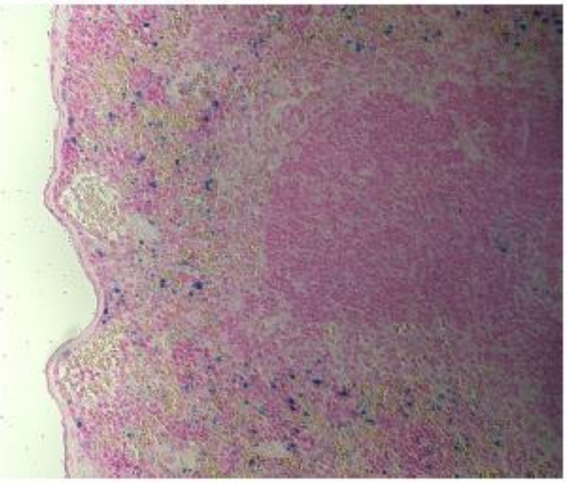

6.85 Gy + Vehicle, 7 days

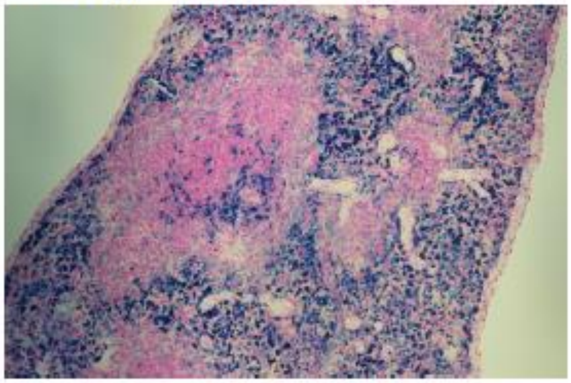

6.85 Gy, + Captopril 7 days

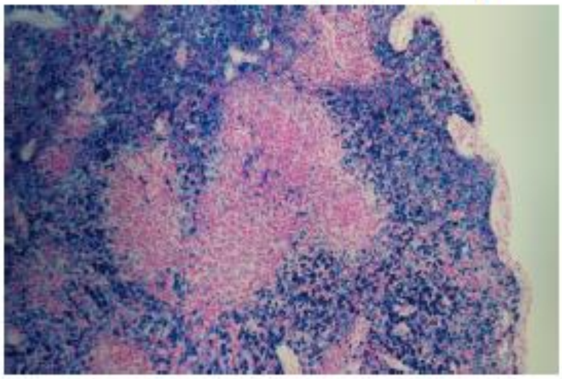

6.85 Gy + Vehicle, 14 days

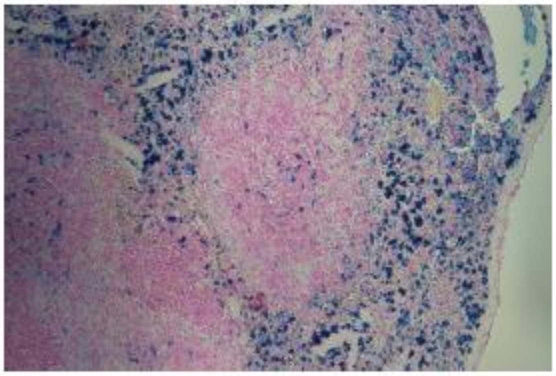

6.85 Gy, + Captopril 14 days

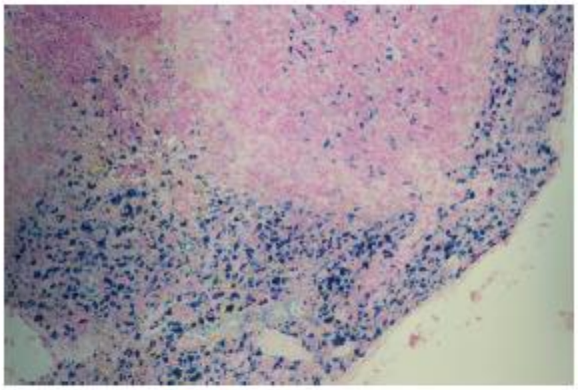

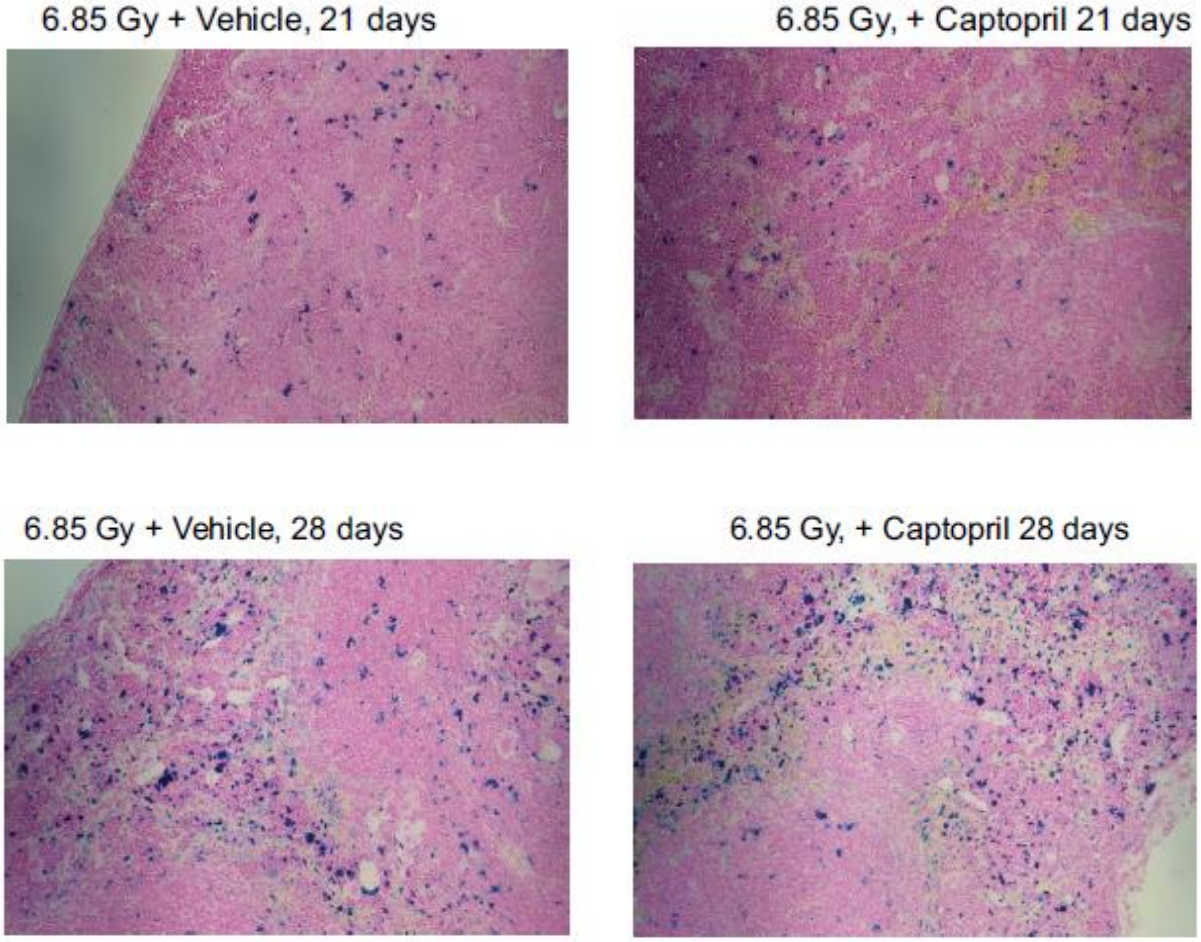

Figure S1: Prussian blue staining of the spleen following total body irradiation. C57BL/6 mice were exposed to 6.85 Gy total body irradiation. Animals received vehicle (drinking water alone) or captopril in the drinking water from days +2 - +16 post-irradiation. Sham mice were treated the same as irradiated mice, but without irradiation; these mice were treated with vehicle. Mice were euthanized at the indicated time points (days post-irradiation) and spleen tissue was obtained for Prussian blue staining. Representative images are shown, 20× magnification.

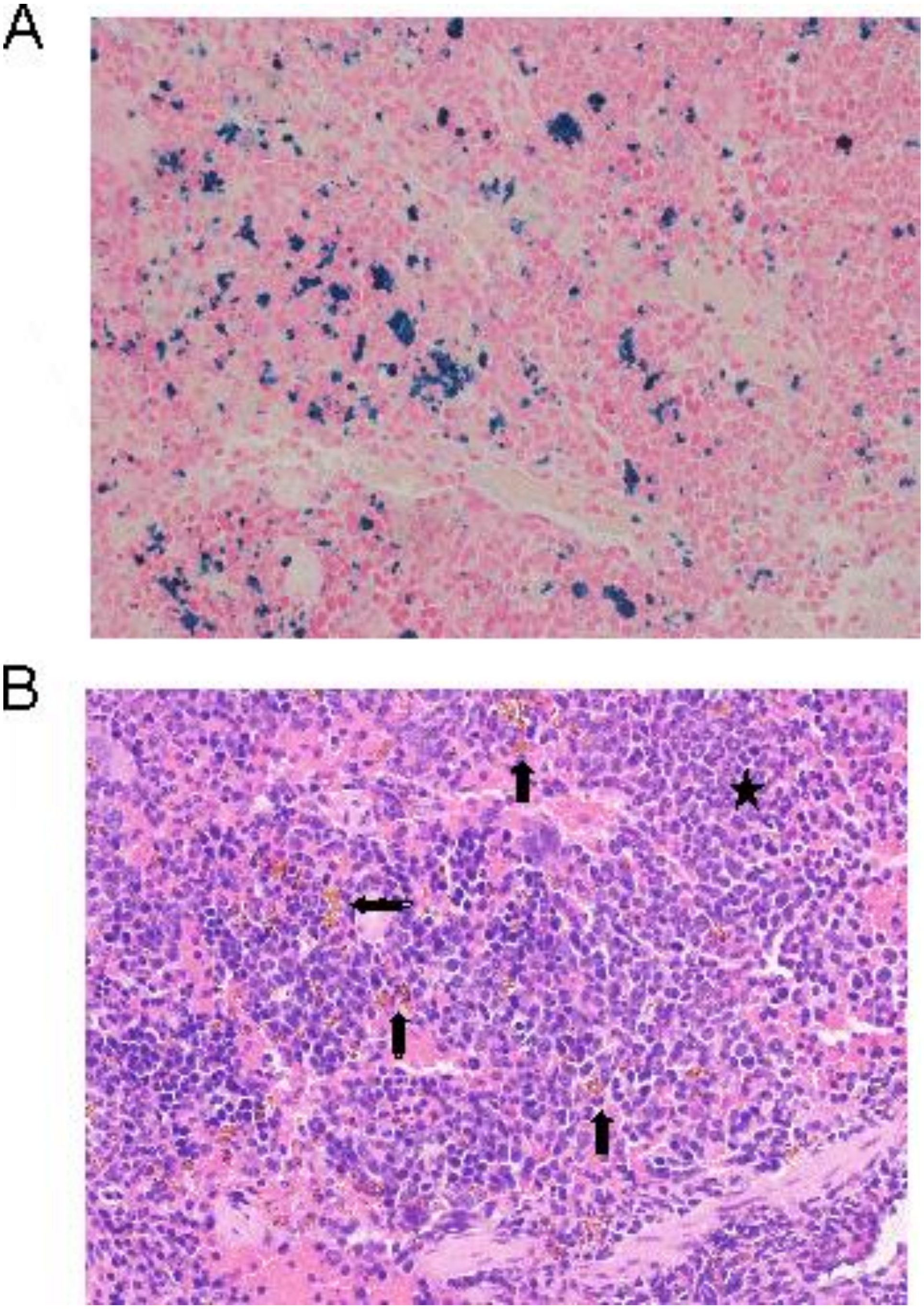

Figure S2: Prussian blue staining and hemosiderin in macrophages following total body irradiation. C57BL/6 mice were exposed to 6.85 Gy total body irradiation. Mice were euthanized at 21 days post-irradiation and spleen tissue was obtained for histology. A. Prussian blue stain with numerous dark blue irregular granules tightly associated with round to oval nuclei of macrophages. B. Hematoxylin & eosin stain section of the same region shown in Figure 1. Yellow staining hemosiderin within macrophage cytoplasm are indicated by black arrows. Also present are mild numbers of neutrophils (black star). Representative images are shown, 400x magnification.

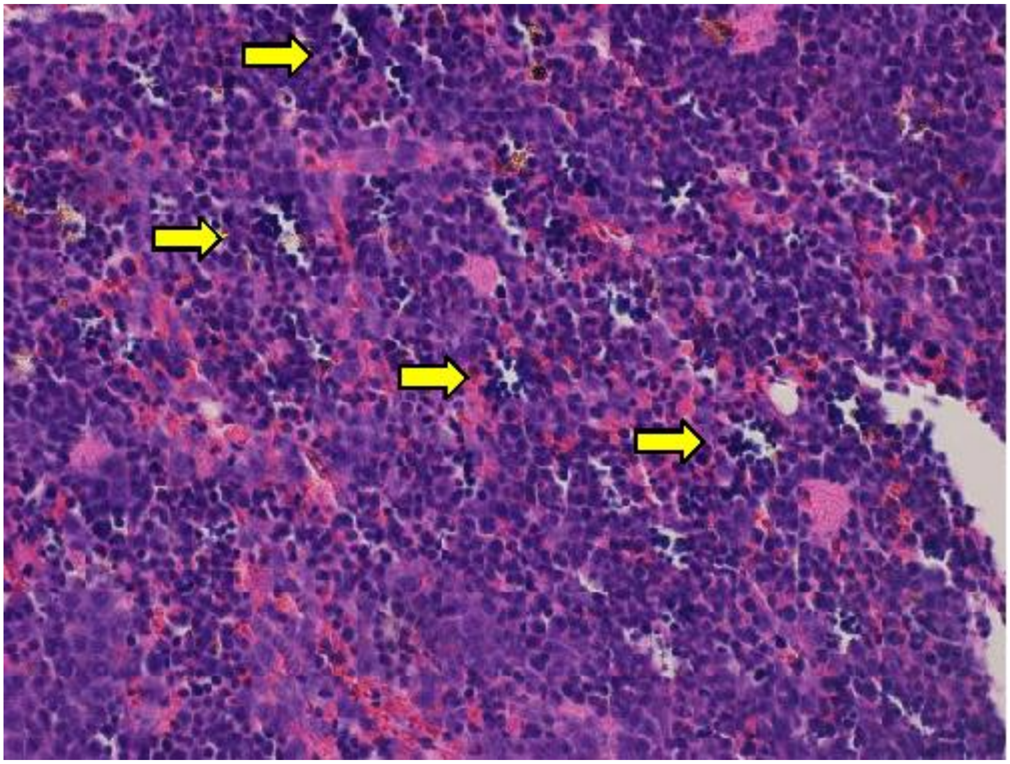

Figure S3: Early stage RBC in the spleen following total body irradiation. C57BL/6 mice were exposed to 6.85 Gy total body irradiation. Mice were euthanized at 21 days post-irradiation and spleen tissue was obtained for histology. Hematoxylin & eosin stain was used to for analysis by a pathologist blinded to the treatment groups. Arrows indicate areas of metarubricyte aggregates. A representative image is shown, 400x magnification.

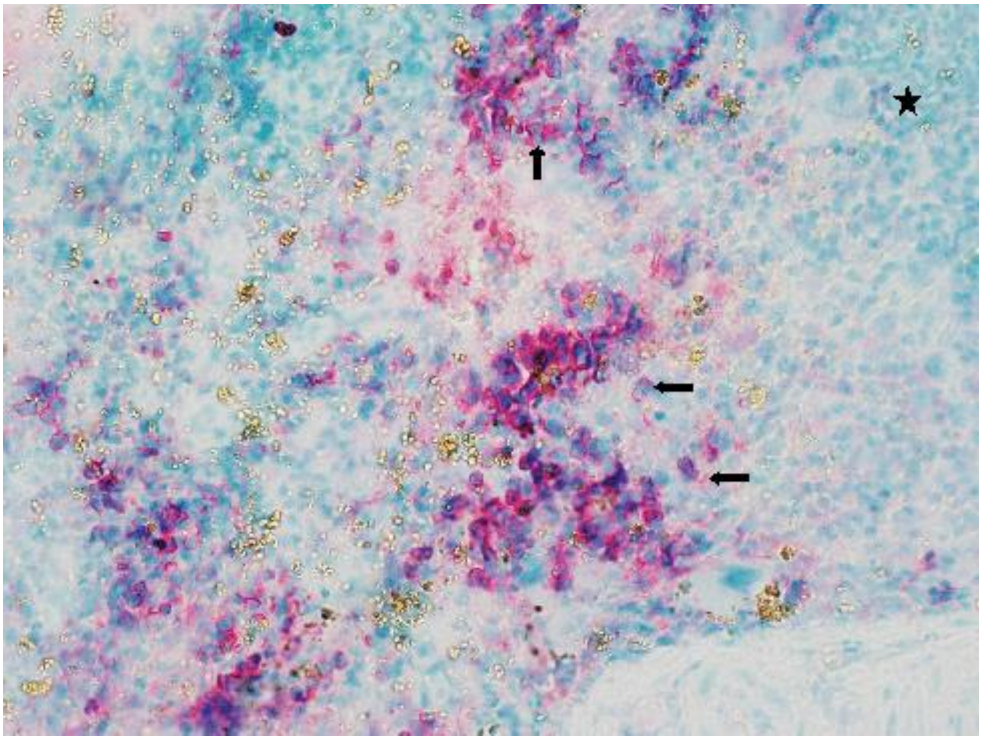

Figure S4: Staining of transferrin receptor/CD71 in the spleen following total body irradiation. C57BL/6 mice were exposed to 6.85 Gy total body irradiation. Mice were euthanized at 21 days post-irradiation and spleen tissue was obtained for histology. Tissue was stained for transferrin receptor/CD71. There is CD71 staining for cells expanding the red pulp indicated by black arrows. The white pulp is marked with a black star as reference. A representative image is shown, 400x magnification.

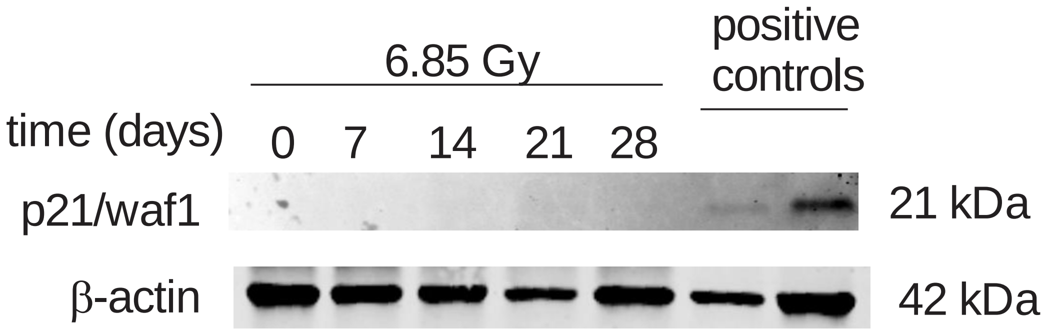

Figure S5: Total body irradiation does not induce markers of accelerated senescence in the spleen. C57BL/6 mice were exposed to 6.85 Gy total body irradiation. Mice were euthanized at the indicated time points and spleen tissue was obtained. Protein lysates were made and used for western blotting for p21/waf , a cell cycle inhibitor found to be elevated in senescent cells. Blots were reprobed for b-actin. Lysate with increased expression of p21/waf1 is included as a positive control. Representative data are shown. The data indicate that p21/waf1 is not upregulated in the spleen following 6.85 Gy total body irradiation at the time points examined.

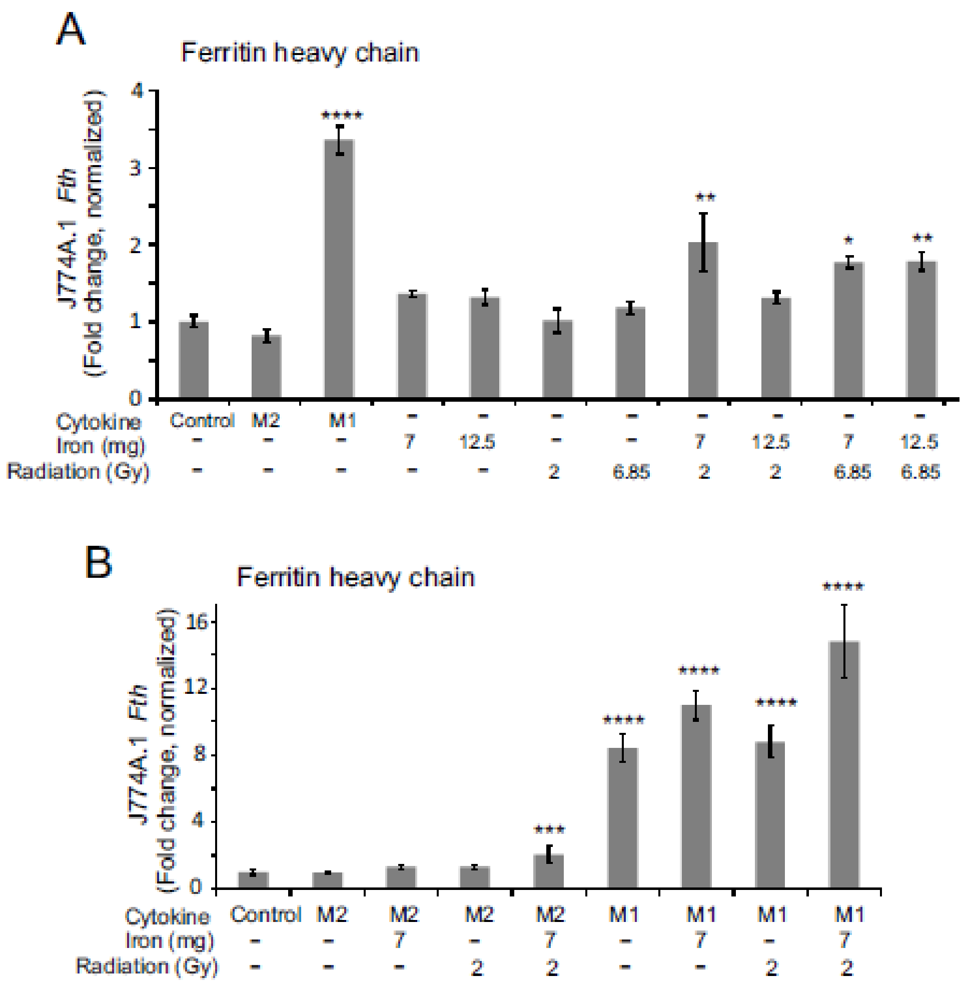

Figure S6: Treatment with IFN-γ and LPS induce ferritin gene expression in J774A.1 murine macrophages in culture. Murine macrophages were grown to 70-85% confluence. A. Cells were exposed to either 2 or 6.85 Gy X-ray irradiation ± 7 or 12 mg/L Fe^{3+}, or 7 or 12 mg/L Fe^{3+} alone. After 24 h, cellular RNA was purified and used for qRT-PCR for Fth1. As contols, cells were treated with IL-4 (100 ng/ml) to induce M2 polarity or IFN-γ (100 ng/ml) + LPS (100 ng/ml) to induce M1 polarity B. Cells were exposed to 2 Gy X-ray irradiation ± 7 mg/L Fe^{3+}, or 7 mg/L Fe^{3+} alone. At 1h after radiation and/or the addition of Fe3+, cells were treated wtih IL-4 (100 ng/ml) to induce M2 polarity or IFN-γ (100 ng/ml) + LPS (100 ng/ml) to induce M1 polarity. Data show means ± SEM, n=3, normalized to the expression of GAPDH. * indicates $p<0.05$ from control; ** indicates $p<0.01$ from control; *** indicates $p<0.001$ from control; **** indicates $p<0.0001$ from control. Data indicate that Fth1 gene expression is increased in response to IFN-g/LPS (M1 polarizing) treatment. Iron + radiation also increased Fth1 expression compared to control in most cases. Iron + radiation increased Fth1 gene expression in the presence of either the M1 or M2 polarizing cytokines. The addition of 2 Gy X-ray irradiation or 7 mg/L to IFN-γ/LPS treatment did not result in significant enhancement of Fth1 expression. In contrast, western blot data showed that ferritin protein was not increased in the absence of the addition of Fe^{3+} to the medium (Fig.11A).

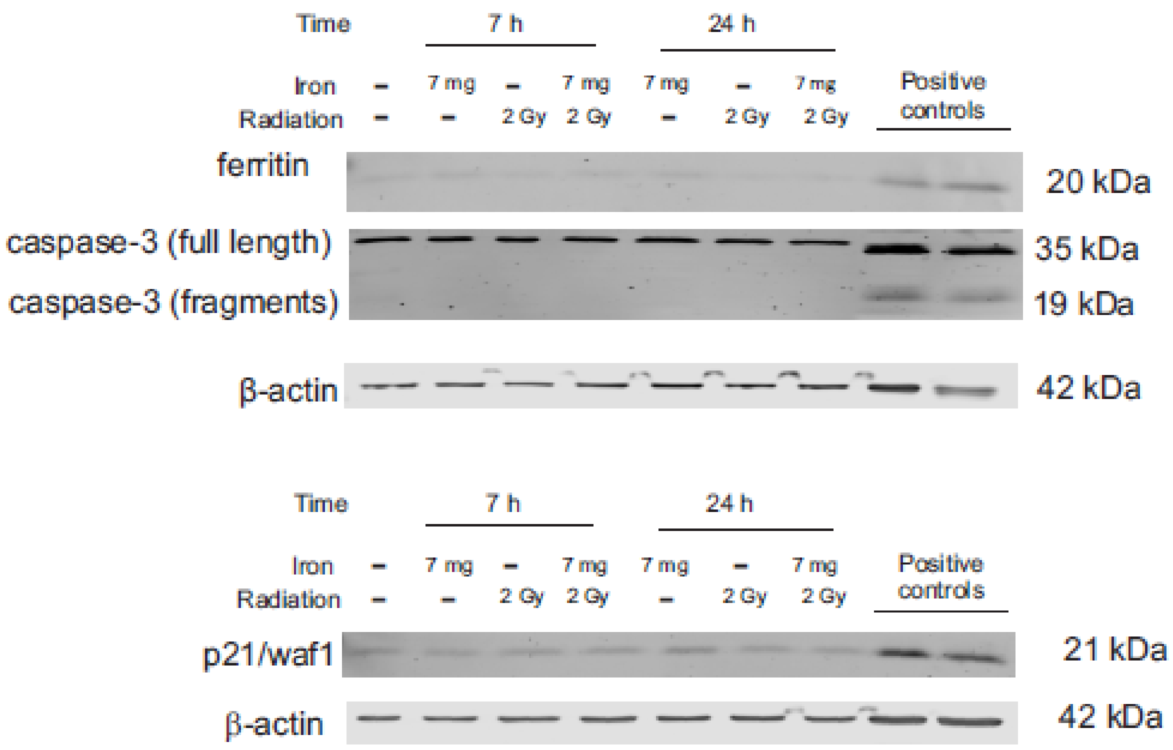

Figure S7: Human spleen microvascular endothelial cells do display increased ferritin or p21/waf1 expression or increased caspase-3 activation following exposure to 2 Gy X-ray irradiation or 7 mg/L Fe^{3+}. HSpMVEC were grown to 70-90% confluence and exposed to either 2 Gy X-ray irradiation and/or 7 mg/L F^{e3+} for 7 h or 12 h. Positive controls were included in each gel for ferritin, cleaved caspase-3, and p21/waf1. Cells were placed on ice, and cell lysates were prepared for western blotting for ferritin, p21/waf1, or cleaved caspase 3. Blots were also probed for β-actin as a loading control. Data show that treatment with iron, radiation, or iron + radiation did not upregulate ferritin, caspase-3 activation, or p21/waf1. The data show that neither iron exposure nor X-ray irradiation cause upregulation of ferritin or the activation of the programmed cell death marker caspase-3 or the accelerated senescence marker p21/waf1 in HSpMVEC.
